# Experimental data on the properties of polymer-modified cement grouts using epoxy and acrylic resin emulsions

**DOI:** 10.1016/j.dib.2016.09.016

**Published:** 2016-09-17

**Authors:** Costas A. Anagnostopoulos, Minas Tsiatis

**Affiliations:** Department of Civil Engineering, School of Technological Applications, Alexander Technological Educational Institute of Thessaloniki, 57400 Thessaloniki, Greece

**Keywords:** Epoxy resin, Acrylic resin, Cement grouts

## Abstract

The use of additives to improve the quality of cement grouts is crucial for civil engineering, especially in foundation construction. This article presents experimental data concerning the compressive strength, elastic modulus, bleeding and injectability of microfine cement grouts modified with epoxy and acrylic resin emulsions. Strength properties were obtained at different curing ages. For further analysis and detailed discussion of properties of polymer-modified cement grouts, see “Fundamental properties of epoxy resin-modified cement grouts” (C.A. Anagnostopoulos, G. Sapidis, E. Papastergiadis, 2016) [1].

**Specifications Table**

**Table:** 

Subject area	*Materials*
More specific subject area	*Polymer modified cement grouts.*
Type of data	*Tables, figures.*
How data was acquired	*Laboratory tests and collection.*
Data format	*Raw, calculated, analyzed, tabulated, plotted.*
Experimental factors	*The specimens of un-modified and polymer- modified cement grouts (PMGs) were prepared and treated as described in*[1].
Experimental features	*Testing the compressive strength and elastic modulus of PMGs with different epoxy and acrylic resin content at designed curing ages in laboratory condition. Rheological measurements were taken from injection tests on soil columns.*
Data source location	*Faculty of Civil Engineering in Thessaloniki, Greece.*
Data accessibility	*Data is with the article.*

**Value of the data**

• This data can be useful for comparing some properties of PMGs with that of ordinary grouts.
• The data highlights the influence of different polymer additives on some properties of cement grouts.
• This article will serve a as guideline to select parameters of PMGs in the development of further research (for instance: type of cement, epoxy resin content, curing time, combination with other additives).

## 1 Data

Composition of the tested grouts (Table 1) and data concerning their strength (Table 2), rheological parameters (Table 3 and Fig. 2) and bleeding (Table 4), collected from authors’ experiments, are presented.

## 2 Experimental design, materials and methods

### 2.1 Materials

The experiments were carried out using a common type of Portland cement (CEM I 52.5 N). A polycarboxylate ether-type (PCE) high range water reducer was used as superplasticiser [2]. Epoxy and acrylic resin emulsions were used as polymer additives. Acrylic resin (AR) is an emulsion of methyl methacrylate-acrylic acid copolymer. Epoxy resin (ER) is water soluble and composed of two components: epoxy resin based on diglycidyl ether of bisphenol-A and an aliphatic amine-based hardener.

### 2.2 Methods

Grouts were prepared with w/c ratios of 0.5, 0.4 and 0.33. The superplasticiser dosage (by cement mass) for the various grouts corresponded to the saturation dosage [3]. The design details of mixtures are presented in Table 1.

Mixing of the grouts was accomplished using a high rotating mixer recommended in ASTM C938-10. In the case of ER-modified grouts, initially, appropriate amounts of cement, water and superplasticiser were thoroughly mixed for 5 min. Afterwards, the required amount of ER, whose two components were mixed in a separate container, was added to the grout, and the resulting mixture was blended for a few minutes to achieve a uniform mixture. Conversely, the preparation of AR-modified grouts was performed by simultaneously mixing cement, water, superplasticiser and acrylic latex.

Bleeding was measured by conducting sedimentation tests according to ASTM C940-10.

Strength development was evaluated from compression tests on specimens cured for 3, 7 and 30 days. Compression tests were performed on cubic specimens with an edge of 50.8 mm at an axial strain of 0.1%/min. The elastic modulus was calculated from the elastic part of the compressive stress-strain curve according to ASTM C469-10. Storage and curing of the specimens followed the suggestions provided by ASTM C109-12.

**Fig. 1:**
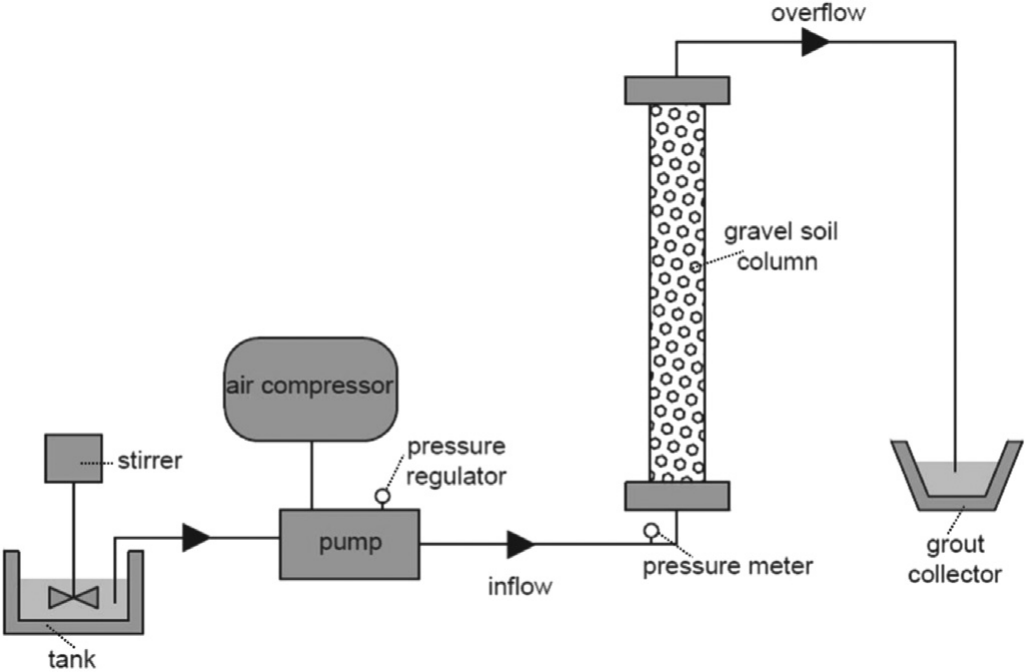
Testing apparatus for the injection experiments.

The effect of polymer type and dosage on the injectability of grouts was evaluated by performing injections into soil columns. The laboratory injection system used in this study was constructed according to ASTM D 4320-04 specification, which allows the adequate simulation of the injection process in the laboratory (Fig. 1). It comprises a mixing tank with a high speed rotating stirrer, an air-operated diaphragm pump, an air compressor, a pressure regulator and pressure meters, plastic molds 100 mm wide, 1500 mm high and 3 mm thick, and the relevant connections. Injection tests were carried out on a gravel soil. The soil had particle size distribution of 4.76–2.38 and a relative density of about 50%. To prepare the specimens, soil samples were poured in the tube in multiple equal layers. Each layer was slightly compacted using a wooden tamp to achieve the desired relative density before placing the next layer. After placing the specimen at the achievable relative density, the top and bottom end-plates of the molds were clamped using tie rods. All grouts were prepared using a high speed rotating mixer and they were continuously agitated to avoid sedimentation of cement particles during the injection tests. The grouts were injected from the bottom of the soil column to produce a more uniform flow of the grouts and avoid any fingering effects that can result in top-to-bottom flow. Injection tests were carried out at a constant pressure of 2 bar. Injection stopped when no flow of grout from the outlet hose of soil column was observed; a consequence of the filtration or clogging mechanism developed inside soil mass during grouting. For the evaluation of the injectability, the total volume of the grout that had passed and collected during the injection was measured, as well as, the flow rates during grouting.

### 2.3 Effect of polymers on strength parameters

Strength parameters of un-modified and polymer-modified cement grouts are presented in Table 2. All of the tests revealed that the addition of polymer additives remarkably increased the 30-day compressive strength and elastic modulus.

### 2.4 Effect of polymers on injectability

**Fig. 2:**
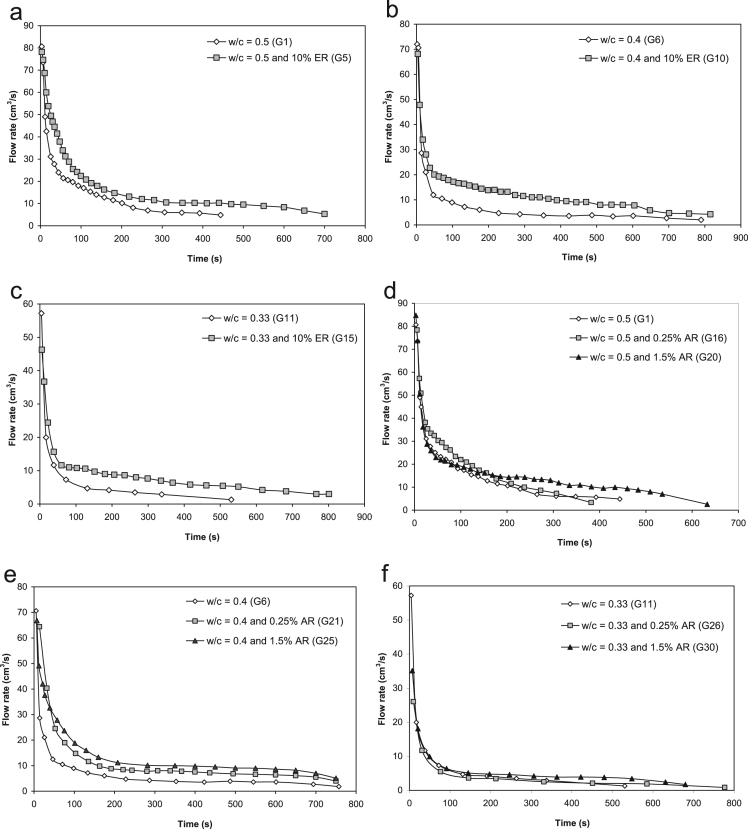
Flow rate during the injection experiments of grouts proportioned with a) w/c=0.5 containing 0 and 10% ER; (b) w/c=0.4 containing 0 and 10% ER; (c) w/c=0.33 containing 0 and 10% ER; (d) w/c=0.5 containing 0, 0.25 and 1.5% AR; (e) w/c=0.4 containing 0, 0.25 and 1.5% AR; and (f) w/c=0.33 containing 0, 0.25 and 1.5% AR.

Table 3 lists the total volume that had passed and Fig. 2 presents the flow rate during the injection experiments of some grouts. Experiments showed that the addition of polymers substantially increased the injectability of all of the grouts.

### 2.5 Effect of polymers on bleeding

The bleeding of grouts in the presence of different dosages of polymer additives was determined and compared (Table 4). Polymer-modified grouts appeared to have volume loss that was higher than the un-modified grouts. This has to be considered when the bleeding of the grout is essential for the purpose of the construction project.

## Figures and Tables

**Table 1:** Composition of the tested grouts.

Notation	Proportion (w/c)	PCE (%)	ER (%)	AR (%)
G1	0.5	0.5	0	
G2			2.5	
G3			5	
G4			7.5	
G5			10	

G6	0.4	1	0	
G7			2.5	
G8			5	
G9			7.5	
G10			10	

G11	0.33	1.5	0	
G12			2.5	
G13			5	
G14			7.5	
G15			10	

G16	0.5	0.5		0.25
G17				0.5
G18				0.75
G19				1
G20				1.5

G21	0.4	1		0.25
G22				0.5
G23				0.75
G24				1
G25				1.5

G26	0.33	1.5		0.25
G27				0.5
G28				0.75
G29				1
G30				1.5

**Table 2:** Development of strength parameters of grouts used in the tests.

Notation	Compressive strength (MPa)			Elastic modulus (GPa)		
	Curing time (days)			Curing time (days)		
	3	7	30	3	7	30
G1	34.2	40.1	48.4	4.4	4.8	5.2
G2	34.5	41.2	50.2	4.8	5	5.9
G3	33.7	44.3	51.3	4.3	5.2	6
G4	33.8	48.2	54.3	4.1	5.5	6.2
G5	33.4	52.7	58.6	4	6.1	6.4
G6	55.2	60	69.2	6.2	6.5	7.2
G7	61.8	66.6	86.4	7	7.7	9.4
G8	53.8	70.3	90.4	5.9	9.1	9.8
G9	58.9	84.2	102.4	6.4	9.5	10.4
G10	66.8	89.1	112.8	8.3	10	11.2
G11	62.9	73.6	90.6	6.4	7.2	9.4
G12	66	76.6	92	7.4	8.4	9.6
G13	72	78	98.7	7.9	8.9	10.1
G14	79.1	94.6	109	8.5	9.8	10.7
G15	83.3	104.5	121	9.1	10.2	11.4
G16	33	38.6	60.5	3.5	4.6	6.4
G17	29	41.5	55.9	4	4.7	6.2
G18	28.3	42.3	51.5	4.6	4.8	6.5
G19	35.5	43.6	50.4	5.1	5.6	6.5
G20	33.7	45.8	52.2	5.3	6.3	6.6
G21	60.5	66	85.2	6.8	7.3	8.6
G22	54	68.5	90.6	7.1	7.9	8.8
G23	59.1	70	94	7.2	8.2	9
G24	68.1	72.3	98.5	7.7	8.6	9.2
G25	64.9	78.9	104.7	7.6	9.2	11
G26	71	80	95.1	7.5	8.3	9.4
G27	70	76.4	102.7	7.7	8.8	10.5
G28	68.3	78.8	104.5	7.9	9.2	10.8
G29	66	76	106	8	9.4	11
G30	64.5	73	109	8.2	9.6	11.3

**Table 3:** Total volume of passed grout obtained from the injection experiments.

Notation	Volume (l)
G1	5.8
G5	11
G6	4.5
G10	9
G11	2.3
G15	5.9
G16	6
G20	8.5
G21	5.5
G25	6.5
G26	2.2
G30	3.1

**Table 4:** Bleeding of grouts.

Notation	Bleeding (%)
G1	4.7
G2	9
G3	9.2
G4	7.9
G5	6.8
G6	2.5
G7	10.4
G8	13.8
G9	16.7
G10	22.5
G11	2.2
G12	4.7
G13	9.7
G14	15.3
G15	18.8
G16	8.5
G17	13.6
G18	15.3
G19	11
G20	9.7
G21	9.6
G22	11.6
G23	11
G24	13.1
G25	12.7
G26	4.6
G27	5.7
G28	6.6
G29	5.9
G30	4.7
